# Small ORFs as New Regulators of Pri-miRNAs and miRNAs Expression in *Human* and *Drosophila*

**DOI:** 10.3390/ijms23105764

**Published:** 2022-05-20

**Authors:** Christine Dozier, Audrey Montigny, Mireia Viladrich, Raphael Culerrier, Jean-Philippe Combier, Arnaud Besson, Serge Plaza

**Affiliations:** 1 Molecular, Cellular and Developmental Biology Department (MCD), Centre de Biologie Integrative (CBI), University of Toulouse, CNRS, UPS, 31062 Toulouse, France; raphael.culerrier@univ-tlse3.fr (R.C.); arnaud.besson@univ-tlse3.fr (A.B.); 2 Laboratoire de Recherche en Sciences Végétales, UMR5546 CNRS, UPS Université de Toulouse, INP, 31320 Auzeville-Tolosan, France; audrey.montigny@gmail.com (A.M.); mireia.viladrich@hotmail.es (M.V.); combier@lrsv.ups-tlse.fr (J.-P.C.)

**Keywords:** smORFs, pri-miRNA, miRNA

## Abstract

MicroRNAs (miRNAs) are small regulatory non-coding RNAs, resulting from the cleavage of long primary transcripts (pri-miRNAs) in the nucleus by the Microprocessor complex generating precursors (pre-miRNAs) that are then exported to the cytoplasm and processed into mature miRNAs. Some miRNAs are hosted in pri-miRNAs annotated as long non-coding RNAs (lncRNAs) and defined as MIRHGs (for miRNA Host Genes). However, several lnc pri-miRNAs contain translatable small open reading frames (smORFs). If smORFs present within lncRNAs can encode functional small peptides, they can also constitute *cis*-regulatory elements involved in lncRNA decay. Here, we investigated the possible involvement of smORFs in the regulation of lnc pri-miRNAs in *Human* and *Drosophila*, focusing on pri-miRNAs previously shown to contain translatable smORFs. We show that smORFs regulate the expression levels of *human* pri-miR-155 and pri-miR-497, and *Drosophila* pri-miR-8 and pri-miR-14, and also affect the expression and activity of their associated miRNAs. This smORF-dependent regulation is independent of the nucleotidic and amino acidic sequences of the smORFs and is sensitive to the ribosome-stalling drug cycloheximide, suggesting the involvement of translational events. This study identifies smORFs as new *cis*-acting elements involved in the regulation of pri-miRNAs and miRNAs expression, in both *Human* and *Drosophila melanogaster*.

## 1. Introduction

MicroRNAs (miRNAs) are small single-stranded non-coding RNAs of approximately 21–22 nt long, which are used by various organisms to regulate growth, cellular homeostasis and development [1]. They mediate post-transcriptional gene silencing by binding to target mRNAs, repressing their translation and/or promoting their degradation. Recently, miRNAs have also been found to act on promoters and enhancers to modulate gene expression [2]. Abnormal expression of miRNAs is implicated in the pathogenesis of several human diseases, such as cancer, as well as neurological, cardiovascular and metabolic diseases [3,4], indicating that a precise dosage and regulation are crucial for homeostasis.

MiRNAs are generated from the cleavage of long primary transcripts (pri-miRNAs) in the nucleus by the Microprocessor complex, which contains the RNase III enzyme DROSHA and its cofactor DGCR8, to generate precursors (pre-miRNAs) of ~ 60–80 nt in length. Pre-miRNAs are then exported to the cytoplasm by Exportin 5 to be processed by the RNase III Dicer into mature miRNAs [1].

Most pri-miRNAs are structurally similar to mRNAs. They are transcribed by RNA polymerase II (Pol II), capped, spliced and polyadenalyted [5]. More recently, genome-wide annotations of pri-miRNA transcript structures in mice and humans have uncovered novel and complex regulatory mechanisms influencing miRNA biogenesis [6]. Indeed, while many miRNAs are located within protein-coding genes, some of them are hosted in RNAs annotated as long non-coding (lncRNAs) and defined as MIRHGs (for miRNA Host Genes). Moreover, many pri-miRNAs exhibit complex gene structures and are expressed as multiple transcript variants, due to alternative promoter usage and/or alternative splicing [6].

Control of miRNA expression occurs both at the transcriptional and post-transcriptional levels. MiRNA levels depend predominantly from pri-miRNA processing efficiency. Pri-miRNA processing is modulated by various factors, including chromatin structure or chromatin re-modeling complexes within pri-miRNA genomic loci [7,8], sequence or structural features of the pri-miRNA [9,10,11], RNA modifications, such as RNA editing and RNA methylation [12,13,14], pri-miRNA and Microprocessor binding factors [15], as well as DROSHA and DGCR8 expression, mutation and activity [16,17].

MiRNA levels also depend on pri-miRNA degradation. Similar to mRNAs, pri-miRNAs are polyadenylated and studies have shown that some of them are subjected to degradation by the PPD (PABPN1 and PAPα/γ-mediated RNA decay) nuclear RNA decay pathway [18]. This pathway involves the poly(A) binding protein PABPN1, the poly(A) polymerase PAP and the nuclear exosome complex. However, other factors can influence pri-miRNA degradation. For instance, the protein Ars2 (Arsenite resistance protein 2) is necessary for the stability of a subset of pri-miRNAs in proliferating cells by interacting with the nuclear Cap-Binding Complex (CBC), which binds to the 5’ cap structure of Pol II transcripts and protects them from de-capping and decay [19].

*Cis*-acting sequences within RNA transcripts can also impact RNA stability. For example, a *cis*-element, called the expression and nuclear retention element (ENE), protects some viral and cellular lncRNAs from rapid nuclear RNA decay through triple helix formation with the poly(A) tail [20,21]. The small open reading frames (smORFs) can also constitute *cis*-acting regulatory elements involved in RNA stability. Indeed, many upstream ORFs (uORFs) located in the 5’UTR of mRNAs, upstream of the protein-coding main ORFs (mORFs), present signatures of active translation [22]. By sequestering ribosomes, uORFs can not only repress translation of the mORFs, but also trigger mRNA degradation by an evolutionarily conserved translation-dependent mechanism called NMD (nonsense-mediated mRNA decay) [23,24,25]. Many cytoplasmic lncRNAs also appear to be targeted by NMD, depending on the presence of smORFs in their sequence [26]. This discovery is consistent with studies showing that this class of transcripts, initially thought to be devoid of coding potential, because of the lack of long ORFs, were found associated with ribosomes [27,28,29]. For several of them, their smORFs-encoded polypeptides were detected, revealing an efficient translation [30,31]. Moreover, some of them were also bound by UPF1 [32], a key actor of the NMD pathway [33], suggesting the implication of smORFs in lncRNA decay. The translation process of lncRNA smORFs was proposed to mimic a transcript with a long 3’UTR, which constitutes an NMD-inducing feature [34]. Thereby, smORFs may exhibit dual functions: they can encode functional peptides but also constitute *cis*-regulatory elements involved in mRNAs and lncRNA stability.

Analyses of the *Human* and *Drosophila* lnc pri-miRNAs with ribosome profiling databases [35] reveal that several of them harbor smORFs exhibiting ribosome translation marks [36,37]. Moreover, a few of them have been shown to encode functional peptides [36,38,39,40,41,42,43]. *Human* miRNA host genes (MIRHGs) have been identified as potential NMD targets [26]; however, the function of smORFs in their decay was not addressed.

Here, we questioned the possible involvement of smORFs in the regulation of lnc pri-miRNAs in *Human* and *Drosophila melanogaster*. We focused on *Human* pri-miR-155, pri-miR-497 and *Drosophila* pri-miR8 since we have previously shown that these pri-miRNAs contain translatable smORFs [36,37,42]. We found that smORFs regulate the expression levels of *human* pri-miR-155, pri-miR-497 and *Drosophila* pri-miR-8 and pri-miR-14. Depending on the pri-miRNA studied, smORFs influence the expression of pri-miRNAs positively or negatively and affect the expression and activity of the corresponding miRNAs, suggesting a nuclear regulation mechanism. This regulation appears independent of the nucleotidic and amino acidic sequences of the smORF, but rather relies upon the presence or absence of a smORF. For *Human* pri-miR-497, *Drosophila* pri-miR-8 and pri-miR-14, their levels are sensitive to the ribosome-stalling drug cycloheximide, suggesting the involvement of translational events in this smORF-dependent regulation mechanism.

Thus, in this study we identified smORFs as new *cis*-acting elements involved in the regulation of pri-miRNAs and miRNA expression, in both *Humans* and *flies*.

## 2. Results

### 2.1. smORF51 Regulates the Levels and Activity of Human primiR-155/miR-155

**Figure 1:**
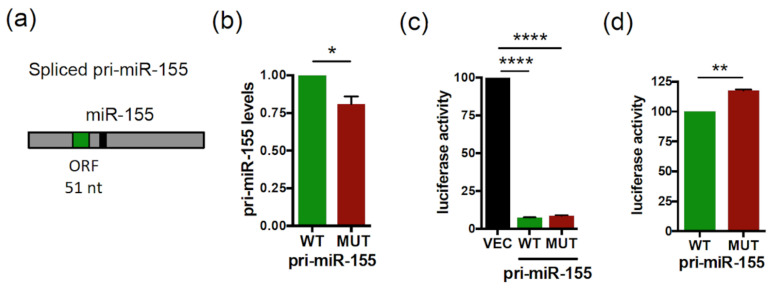
The smORF51 of *Human* pri-miR-155 regulates the expression and activity of pri-miR-155/miR-155. (**a**) Schematic structure of *Human* spliced pri-miR-155 with the miR-155 (black box) and smORF51 (green box) indicated. (**b**) Relative expression levels of wild type (WT) or mutant (MUT) pri-miR-155 transfected in HeLa cells and determined by quantitative RT-PCR analyses (qRT-PCR). Pri-miR-155 levels were normalized to EGFP (encoded in the pri-miRNA expression plasmids, and used to monitor transfection efficiency) and set to 1 for WT pri-miR-155 transfected cells. Data are means ± S.E.M. *n* = 4. (**c**,**d**) Relative activity of miR-155 processed from the WT or mutant pri-miR-155 constructs transfected in HeLa cells together with the luciferase miR-155 sensor. Cells were tested 48 h post-transfection for dual luciferase assays. (**c**) The luciferase activities of pri-miR-155 transfected cells were compared to that of control vector transfected cells (VEC), set to 100. (**d**) The relative luciferase activity of MUT pri-miR-155 was compared to that of WT pri-miR-155, set to 100. Data are means ± S.E.M. *n* = 10. * *p* < 0.05, ** *p* < 0.01, **** *p* < 0.0001.

**Figure 2:**
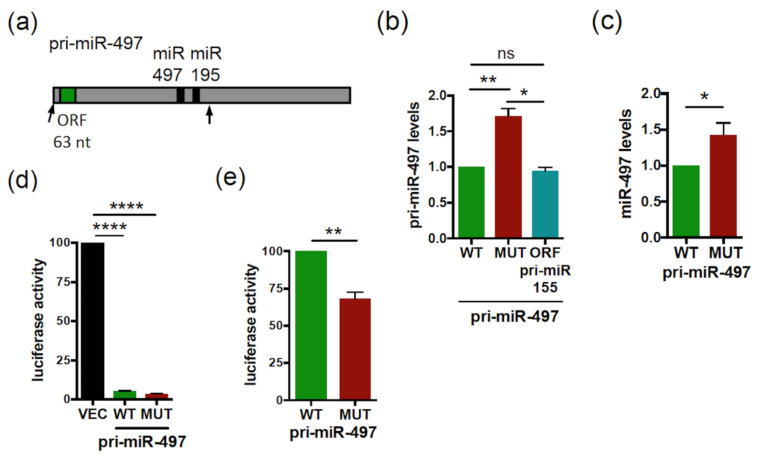
The smORF63 of *Human* pri-miR-497 regulates the expression levels and activity of pri-miR-497/miR-497. (**a**) Schematic structure of *Human* pri-miR-497 with the miR-497 (black box) and smORF63 (green box) indicated. The arrows in the pri-miR-497 delimit the sequences cloned and tested. (**b**) Relative expression levels of pri-miR-497 WT or ATG-mutated (MUT) or with the smORF63 replaced by the smORF51 from pri-miR-155 (ORF pri-miR-155) transfected in Hela cells and determined by qRT-PCR. The pri-miR-497 levels were determined and normalized as in Figure 1b. Data are means ± S.E.M. *n* = 4. (**c**) Relative expression levels of miR-497 processed from WT or MUT pri-miR-497 transfected into Hela cells and determined by qRT-PCR. The miR-497 levels were normalized as in Figure 1b. Data are means ± S.E.M. *n* = 7. (**d**,**e**) Relative activity of miR-497 processed from the WT or MUT pri-miR-497 constructs transfected in Hela cells together with the luciferase miR-497 sensor. Cells were tested 48 h post-transfection for dual luciferase assays. (**d**) The luciferase activities of pri-miR-497 transfected cells were compared to that of vector transfected cells (VEC), set to 100. (**e**) The relative luciferase activity of MUT pri-miR-497 was compared to that of WT pri-miR-497 set to 100. Data are means ± S.E.M. *n* = 8. * *p* < 0.05, ** *p* < 0.01, **** *p* < 0.0001, ns: not significant.

We have previously shown that the *Human* pri-miR-155 and pri-miR-497 transcripts contain translatable smORFs of 51 and 63 nt, respectively (Figure 1a and Figure 2a) [37]. Since smORFs were shown to affect lncRNA stability by triggering co-translational RNA decay pathways [26], we investigated whether the translatable smORFs, present in pri-miR-155 and pri-miR-497, were also involved in the regulation of the pri-miRNA expression. To this end, we constructed vectors expressing the spliced pri-miR155 isoform with the wild type (WT) or the ATGs-mutated (MUT) smORF51. We previously showed that mutation of the three ATGs present within smORF51 into TAG abolishes its translatability [37]. RNA fold predictions showed no alteration of the MFE (minimum free energy) secondary structures between the WT and mutated pri-miR-155 (Figure S1a). Transfection of the WT or mutant pri-miR-155 constructs in HeLa cells revealed that while the overexpression of both pri-miR constructs was detected at the RNA levels (Figure S2a), mutant pri-miR-155 was expressed at lower levels than the WT construct (Figure 1b). Since spliced pri-miR-155 can be used for miR-155 processing [44,45], we investigated whether smORF51 mutation would impact the production and activity of mature miR-155 by co-transfecting a luciferase sensor of miR-155 activity, together with a WT or mutant pri-miR-155 construct or a control vector (VEC). Luciferase reporter assays revealed that expression of both pri-miR-155 constructs repressed the miR-155 sensor (Figure 1c), indicating that both WT and mutant spliced pri-miR-155 constructs are processed into functional mature miR-155 in HeLa cells, in agreement with what was previously reported in B-cell lymphomas for the WT spliced pri-miR-155 [44,45]. However, the luciferase activity was significantly increased (~18%) with the mutant pri-miR-155 construct, compared to WT (Figure 1d), indicating a less efficient repressive capacity. Since we previously showed that overexpression of the smORF51-encoded peptide, miPEP155, in HeLa cells has no impact on pri-miR-155 expression and on miR-155 activity [37], these results suggest that the smORF51 of pri-miR-155 positively regulates the expression of pri-miR155 in *cis*, consequently affecting miR-155 activity.

### 2.2. smORF63 Regulates the Levels and Activity of Human pri-miR-497/miR-497

We then tested a possible role of smORF63, present in the pri-miR-497 (Figure 2a), on the regulation of pri-miR-497 expression. For this, vectors expressing the pri-miR-497 transcript, spanning from the 5’ end to 53 nt downstream of miR-195 (delineated by arrows in Figure 2a), with the WT or ATG/TGA-mutated smORF63 were generated. In this mutant transcript, translation of smORF63 is suppressed [37]. Transfection of the WT or mutant pri-miR-497 constructs in Hela cells shows that while the expression of both pri-miR constructs was detected at the RNA levels (Figure S2b), the mutant pri-miR-497 levels were approximately 1.5-fold that of WT (Figure 2b). Quantification of the mature miR-497 levels revealed that both WT and mutant pri-miR-497 constructs are processed into mature miR-497 (Figure S3); however, the processing of mutant pri-miR-497 yielded higher levels of mature miR-497 than WT (Figure 2c). Thus, mutation of smORF63 also affects miR-497 synthesis. In agreement with this, mutant pri-miR-497 was more efficient in repressing a luciferase sensor of miR-497 activity than its WT counterpart in luciferase assays (Figure 2d,e). Considering that miPEP497, encoded by smORF63, does not regulate the levels of its own pri-miRNA and the activity of the processed miR-497 [37], this result suggests that smORF63 acts in *cis* to inhibit the expression of pri-miR-497, thereby affecting miR-497 production and activity. This is not due to an effect on the secondary structure, since predicted MFE secondary structures show no difference between WT and mutant pri-miR-497 (Figure S1b). We next tested whether the sequence of smORF63 was specifically required for this regulation by constructing a pri-miR-497 in which smORF63 was substituted by smORF51 of the pri-miR155. Transfection of this construct in Hela cells showed that pri-miR-497 with a smORF51 substitution behaved as WT pri-miR-497 (Figure 2b), indicating that this regulation does not depend on the nucleotide composition of the smORF, but rather on the presence of a smORF. Since the WT and mutant pri-miR-497 transcripts are expressed from the same promoters, this suggests a post-transcriptional mechanism.

**Figure 3:**
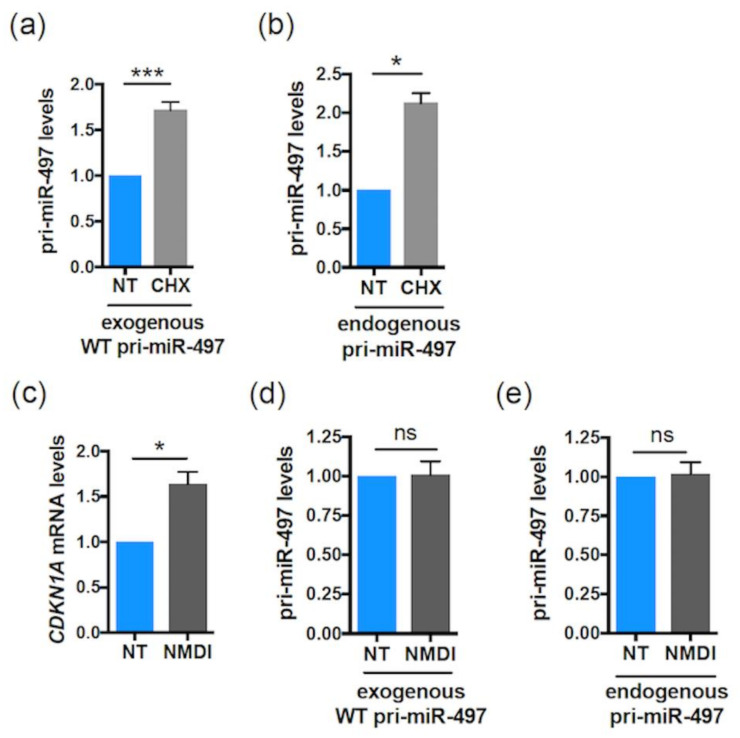
The *Human* pri-miR-497 is sensitive to CHX but not regulated by NMD. (**a**,**b**) Relative expression levels of WT pri-miR-497 transfected in HeLa cells (**a**) or endogenous pri-miR-497 (**b**) upon CHX treatment (50 µg/mL for 1 h). Data are means ± S.E.M. (**a**) *n* = 6, (**b**) *n* = 3. (**c**–**e**) Relative expression levels of CDKN1A mRNA (used as positive control) (**c**) or WT pri-miR-497 transfected in Hela cells (**d**) or endogenous pri-miR-497 (**e**) upon NMDI14 treatment (50 µM for 6 h). Data are means ± S.E.M. *n* = 4. (**a**,**d**) WT pri-miRNAs levels determined by qRT-PCR were normalized to EGFP and set to 1 for the untreated (NT) transfected cells. (**b**,**c**,**e**) The CDKN1A mRNA or endogenous pri-miR-497 levels determined by qRT-PCR were normalized to actin (**b**) or GAPDH (**c**,**e**) and set to 1 for the untreated cells. * *p* < 0.05, *** *p* < 0.0005, ns: not significant.

Since the mutation of smORF63 increases both pri-miR-497 and miR-497 levels, and given that pri-miRNA processing takes place in the nucleus, this suggests that smORFs can regulate pri-miRNA expression in the nucleus. Moreover, the fact that this regulation is smORF-dependent suggests the involvement of ribosomes. This is reminiscent of previous studies showing that mammalian nonsense codons can be *cis* effectors of nuclear mRNA half-life, by reducing the abundance of nuclear mRNAs in a post-transcriptional decay mechanism involving translation by ribosomes [46,47,48,49]. To test whether translational events are involved in the regulation of pri-miR-497 by smORF63, cells transfected with the WT pri-miR-497 were treated with the translation inhibitor cycloheximide (CHX). CHX treatment for 1h induced the accumulation of both exogenous and endogenous pri-miR-497 (Figure 3a,b, respectively). These results suggest a smORF-dependent post-transcriptional mechanism involving translational events. One such mechanism could be NMD, because translation is necessary for NMD, and there is strong evidence for intranuclear NMD [50,51,52]. To test this hypothesis, we used NMDI14, a NMD inhibitor that disrupts the SMG7-UPF1 complex, a key component of the NMD pathway [53]. NMDI treatment caused the accumulation of *CDKN1A* mRNA, a bona fide NMD substrate [54] (Figure 3c), without affecting the levels of pri-miR-497, both exogenous and endogenous (Figure 3d,e, respectively). This suggests that smORF63 regulates pri-miR-497 expression by an NMD-independent mechanism involving translational events.

Altogether, these results suggest that smORFs can act in *cis* to regulate positively or negatively the expression of pri-miRNAs/miRNAs in *Humans*.

### 2.3. Drosophila pri-miR-8 Contains a smORF Regulating Its Expression and Activity

**Figure 4:**
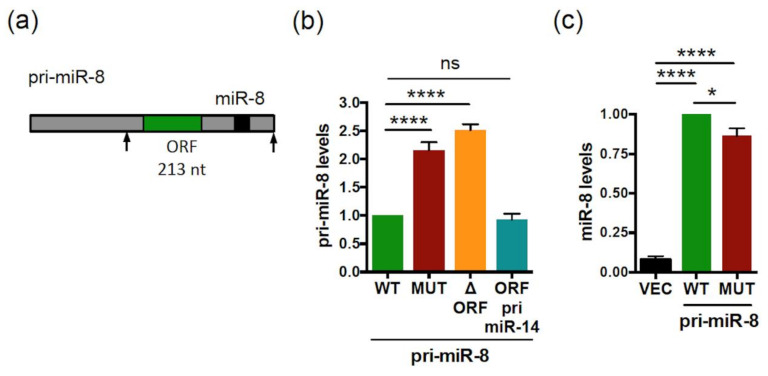
The *Drosophila* pri-miR-8 contains a smORF regulating its expression level. (**a**) Schematic structure of *Drosophila* pri-miR-8 with the miR-8 (black box) and smORF213 (green box) indicated. The arrows in the pri-miR-8 delimit the sequences used for analyses. (**b**) Relative expression levels of pri-miR-8 WT or ATG-mutated (MUT) or deleted of the entire smORF213 (ORF) or with the smORF213 replaced by the smORF225 from pri-miR-14 (ORF pri-miR-14) transfected in S2 cells and determined by qRT-PCR. The pri-miR-8 levels were normalized to *GAL4* (using pActin-GAL4 vector cotransfected with the pri-miRNA expression plasmids and used to monitor transfection efficiency) and set to 1 for the WT pri-miR-8 transfected cells. Data are means ± S.E.M. *n* = 10. (**c**) Relative expression levels of miR-8 from vector (VEC) or WT or MUT pri-miR-8 transfected S2 cells and determined by qRT-PCR. The miR-8 levels were normalized to *U14* and compared to the WT pri-miR-8, set to 1. Data are means ± S.E.M. *n* = 15. * *p* < 0.05, **** *p* < 0.0001, ns: not significant.

We next investigated whether the smORF-dependent pri-miRNA/miRNA regulation identified above also occurs in other species. We chose to address this question in *Drosophila melanogaster*, first focusing on pri-miR-8 that was previously shown to contain a translatable smORF of 213 nt (Figure 4a) [36]. To test a possible role of the smORF213 in the regulation of pri-miR-8 expression, vectors expressing part of the pri-miR-8 transcript, spanning over pre-miR-8 (delineated by arrows in Figure 4a), with the WT or ATG/AGT-mutated smORF213, were generated. We previously showed that the mutant transcript is no longer able to translate the smORF213 [36]. Expression of the WT or mutant pri-miR-8 constructs in *Drosophila* S2 cells revealed that while the expression of both pri-miR constructs was detected (Figure S4), the pri-miR-8 mutant accumulated at twice the levels of the WT construct (Figure 4b). We then tested whether mutation of smORF213 affected miR-8 synthesis. Surprisingly, quantification of mature miR-8 levels indicated that, while both WT and mutant pri-miR-8 constructs are processed into mature miR-8, the mutant pri-miR-8 yielded lower levels of mature miR-8 (Figure 4c).

**Figure 5:**
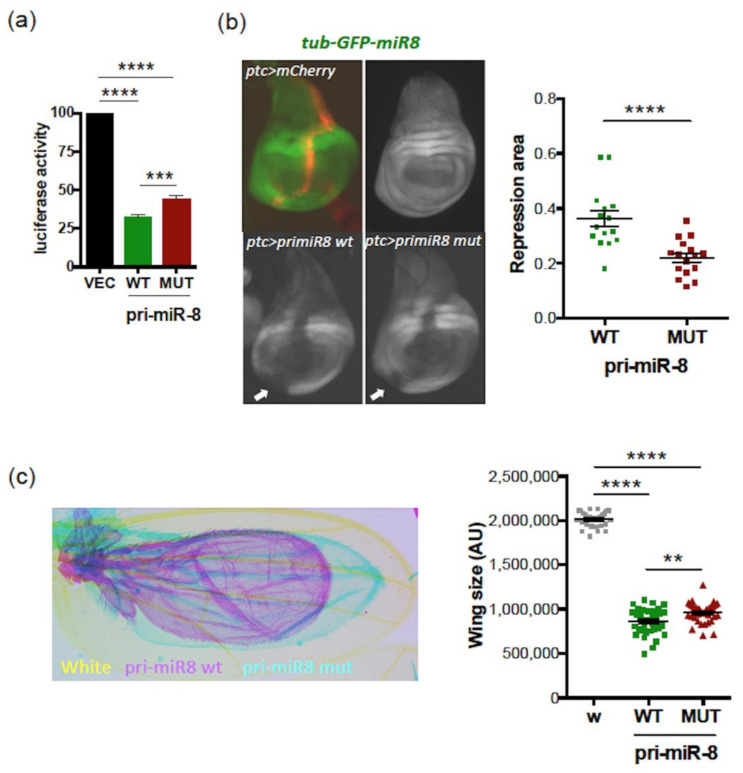
The smORF213 regulates the activity of *Drosophila* pri-miR-8/miR-8. (**a**) Relative activity of miR-8 processed from the WT or MUT pri-miR-8 constructs transfected in S2 cells together with a miR-8 luciferase sensor. Cells were tested 48h post-transfection for dual luciferase assays. The luciferase activities of pri-miR-8 transfected cells were compared to that of control vector transfected cells (VEC), set to 100. Data are means ± S.E.M. *n* = 8. (**b**) Activity of miR-8 processed from the WT or MUT pri-miR-8 in wings imaginal discs. Left panel: representative discs are shown, the pri-miR-8 are expressed under the *patched* (*ptc*) GAL4 promoter and the miR-8 activity is detected with a *tubulin* GFP miR-8 sensor expressed in wing imaginal discs. The *ptc* domain is visualized with mCherry. Right panel: quantification of the repression area (indicated by white arrows in the left panel). Data are means ± S.E.M. from 15 wing discs analyzed for WT pri-miR-8 and 16 for MUT. (**c**) Activity of WT or MUT pri-miR-8 on wing size. The WT or MUT pri-miR-8 constructs were expressed in wings using the MS1096 driver and the phenotypes scored on wing size. Left panel: representative wings are shown. Right panel: quantification of the wing size. AU: Arbitrary Units. Data are means ± S.E.M. from 29 wings analyzed for control (white, w), 34 for WT pri-miR-8 and 35 for MUT. ** *p* < 0.01, *** *p* < 0.001, **** *p* < 0.0001.

Accordingly, luciferase assays, using a luciferase sensor of miR-8 activity in S2 cells, confirmed that both WT and mutant pri-miR-8 constructs were processed into functional miR-8; however, WT pri-miR-8 exhibited more miR-8 activity than its mutant counterpart (Figure 5a). Similar experiments were performed in vivo using a GFP miR-8 sensor expressed under the *tubulin* promoter (*tub-GFP-miR8*) in wing imaginal discs, in which miR-8 is functional [55]. Expression of WT or mutant pri-miR-8 under the *patched* (*ptc*) GAL4 promoter led to the repression of GFP in and outside the *ptc* domain (visualized with mCherry), indicating that miR-8 acts in a non-cell autonomous manner (Figure 5b, left panels). However, the area of the repressed domains was reduced with the mutant pri-miR-8 compared to WT (Figure 5b, right panel). These results were confirmed by generating flies overexpressing WT or mutant pri-miR-8 constructs in wings, using the wing driver line MS1096. As expected, overexpression of these pri-miR-8 constructs induced a “small wing” phenotype (Figure 5c, left panel), as observed upon miR-8 overexpression [36,56]. However, quantifying wing size revealed a weaker activity of the mutant pri-miR-8 compared to WT (Figure 5c, right panel). Since we previously showed that miPEP8, encoded by smORF213, does not regulate the expression of pri-miR-8 and the activity of miR-8 both in S2 cells and in flies [36], our results suggest that smORF213 acts in *cis* to regulate the expression of pri-miR-8. Accordingly, overexpressing the WT or mutant pri-miR-8 in miR-8 expression domains, using a miR-8 GAL4 driver, revealed that mutant pri-miR-8 accumulated at higher levels than its WT counterpart and exhibited lower levels of mature miR-8 (Figure S5).

**Figure 6:**
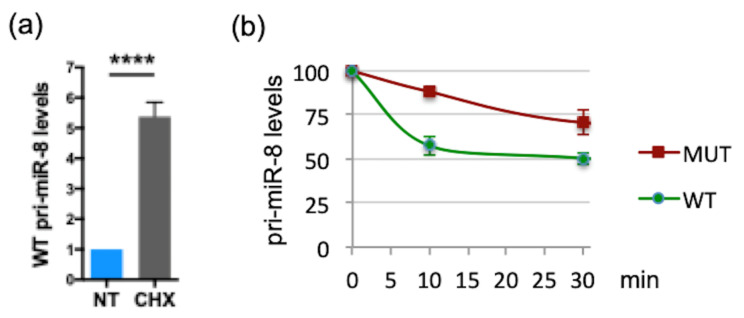

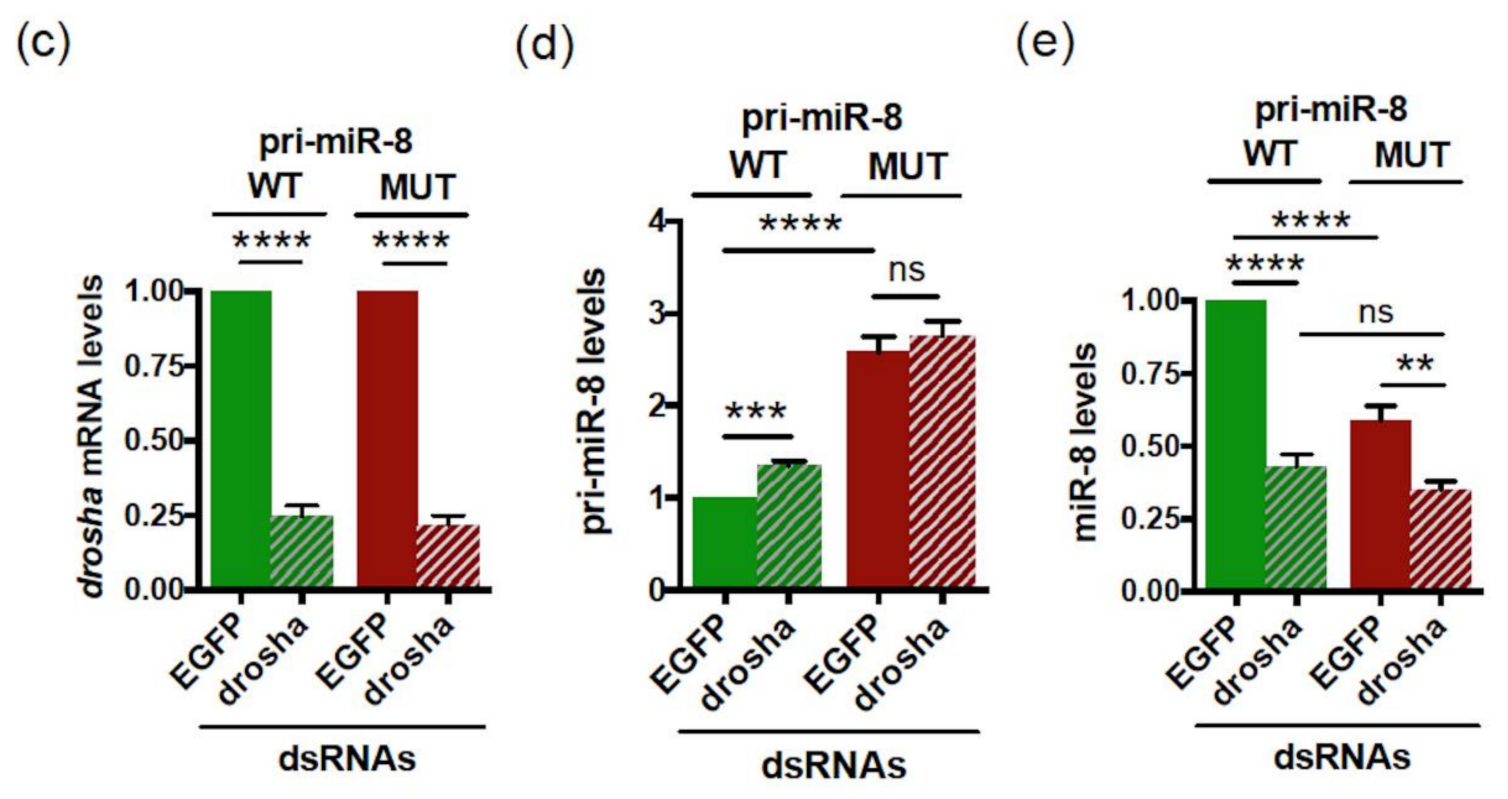
The mutant pri-miR-8 is more stable and insensitive to Drosha depletion. (**a**) Relative expression levels of WT pri-miR-8 transfected into S2 cells upon CHX treatment (30 µg/mL for 4 h). WT pri-miR-8 levels, determined by qRT-PCR, were normalized to *GAL4* and set to 1 for the untreated (NT) transfected cells. Data are means ± S.E.M. *n* = 7. (**b**) Relative expression levels of WT or MUT pri-miR-8 transfected in S2 cells, upon Actinomycin D treatment (1 mg/mL) for various times. Pri-miR-8 levels, determined by qRT-PCR, were normalized to RP49 and set to 100 for the untreated transfected cells. Data are representative of two independent experiments performed in triplicate. (**c**,**d**,**e**) Relative expression levels of *Drosha* mRNA (**c**) or pri-miR-8 (**d**) or miR-8 (**e**) in S2 cells first transfected with dsRNA EGFP or dsRNA drosha and then transfected with WT or MUT pri-miR-8 constructs. (**c**) The *drosha* mRNA levels, determined by qRT-PCR, were normalized to *tubulin* and set to 1 for EGFP dsRNA transfected cells. (**d**,**e**) the pri-miR-8 levels (**d**) and miR-8 levels (**e**) determined by qRT-PCR, were normalized to *GAL4* for pri-miR-8 (**d**) and *U14* for miR-8 (**e**) and set to 1 for EGFP dsRNA WT pri-miR-8 transfected cells. Data are means ± S.E.M. *n* = 8. ** *p* < 0.01, *** *p* < 0.001, **** *p* < 0.0001, ns: not significant.

Similar to pri-miR-497, the coding sequence of smORF213 was not specifically required for this regulation. Indeed, while deleting the entire smORF213 (pri-miR-8 ΔORF) had similar effects as mutating the ATG of this smORF, i.e., accumulation of pri-miR-8 (Figure 4b), replacing smORF213 by smORF225 from pri-miR-14 (pri-miR-8 ORF pri-miR-14) restored pri-miR-8 levels to that of WT (Figure 4b). Nevertheless, the secondary structure of pri-miR-8 ORF pri-miR-14 was substantially altered using RNA fold prediction (Figure S6). This suggests that the differences in pri-miR-8 levels observed with the ATG-mutated and ΔORF pri-miR-8 were not due to altered folding of their secondary structures (Figure S6). Moreover, in all mutants of pri-miR-8, the refolding does not affect their pre-miR-8 secondary structure (Figure S6). Together, these experiments suggest that the function of the smORF is independent of its amino acid sequence to fine-tune miR-8 levels. Since pri-miR-8 regulation requires the presence of a smORF, the involvement of translational events was tested in S2 cells transfected with WT pri-miR-8 treated or not with CHX. Upon CHX treatment, WT pri-miR-8 accumulated (Figure 6a), suggesting a mechanism requiring translation by ribosome. However, the regulatory mechanism triggered by the smORF appears different from that of pri-miR-497. Indeed, with the ATG-mutated pri-miR-8 construct, miR-8 expression was inversely correlated with pri-miR-8, suggesting that the processing of the pri-miRNA could be affected. This step in miRNA biogenesis is carried out in the nucleus by the microprocessor complex that contains the RNase III DROSHA. Depletion of DROSHA impairs pri-miRNA processing, thereby stabilizing pri-miRNA transcripts and increasing pri-miRNAs levels [57]. To test whether processing of the pri-miR-8 mutant is altered, WT or mutant pri-miR-8 transfected cells were treated with the transcription inhibitor actinomycin D for various times, and RNA decay was monitored. In these experiments, the mutant pri-miR8 was more stable than its WT counterpart (Figure 6b). Moreover, if processing of the mutant pri-miR-8 is altered, DROSHA depletion should only have a minor impact on its accumulation. To test this hypothesis, S2 cells were transfected with dsRNA against EGFP (control) or against *drosha*, and with WT or mutant pri-miR-8. *Drosha* dsRNA transfection resulted in efficient reduction of *drosha* mRNA (~75%) (Figure 6c). Confirming the results obtained previously (Figure 4b), the mutant pri-miR-8 accumulated to twice the levels of WT pri-miR-8 in EGFP dsRNA transfected cells (Figure 6d). However, while WT pri-miR-8 increased (~1.35 fold) in *drosha* depleted cells compared to control cells, no significant change in mutant pri-miR-8 levels (~1.06) was observed (Figure 6d). Quantification of mature miR-8 levels showed that mutant pri-miR-8 yielded lower levels of miR-8 than WT pri-miR-8 in EGFP dsRNA transfected cells (Figure 6e), as observed previously (Figure 4c). Nevertheless, upon *drosha* depletion, miR-8 levels processed from WT pri-miR-8 decreased more efficiently (2.3-fold) than from mutant pri-miR-8 (1.6-fold). Altogether, these results suggest that smORFs serve to fine tune pri-miRNA processing.

Thus, as in *Humans*, smORF-dependent pri-miRNA/miRNA regulation also takes place in *Drosophila melanogaster* and appears to involve translational events. However, smORFs differently affect pri-miRNAs and miRNA expression, revealing distinct regulation mechanisms between humans and flies.

### 2.4. smORFs Regulate the Levels and Activity of Drosophila pri-miR-14/miR-14

**Figure 7:**
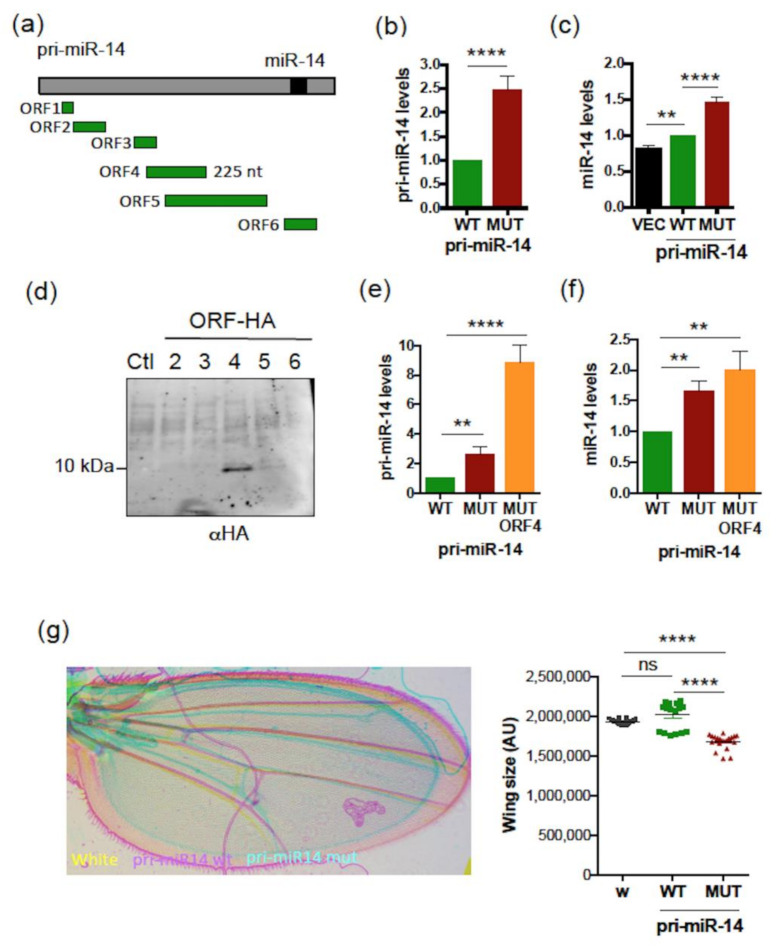
*Drosophila* pri-miR-14 contains smORFs regulating its expression and activity. (**a**) Schematic structure of *Drosophila* pri-miR-14 with the miR-14 (black box) and the six smORFs (green box) indicated. (**b**) Relative expression levels of pri-miR-14 WT or all ATG-mutated (MUT) transfected into S2 cells and determined by qRT-PCR. Pri-miR-14 levels were normalized as in Figure 4b. Data are means ± S.E.M. *n* = 14. (**c**) Relative expression levels of miR-14 from control vector (VEC) or WT or MUT pri-miR-14 transfected S2 cells and determined by qRT-PCR. MiR-14 levels were normalized as in Figure 4c. Data are means ± S.E.M. *n* = 15. (**d**) Translatability of pri-miR-14. Pri-miR-14 constructs with the smORFs fused to the HA tag were translated in vitro and analyzed by western blot. Translation of the smORF was detected with an anti-HA antibody. (**e**) Relative expression levels of WT or MUT or ORF4 ATG-mutated (MUT ORF4) pri-miR-14 transfected in S2 cells and determined by qRT-PCR. The pri-miR-14 levels were normalized as in Figure 4b. Data are means ± S.E.M. *n* = 15. (**f**) Relative expression levels of miR-14 processed from WT or MUT or MUT ORF4 pri-miR-14 transfected in S2 cells and determined byRT-PCR analyses. The miR-14 levels were determined as in Figure 4c. Data are means ± S.E.M. *n* = 10. (**g**) Activity of WT or MUT pri-miR-14 on wing size. The WT or MUT pri-miR-14 constructs were expressed in wings using the MS1096 driver and the phenotypes scored on wing size. Left panel: representative wings are shown. Right panel: quantification of the wing size. AU: Arbitrary Unit. Data are means ± S.E.M. from 19 wings analyzed for control (white, w), 18 for WT pri-miR-14 and 19 for MUT pri-miR-14. ** *p* < 0.01, **** *p* < 0.0001, ns: not significant.

We then tested whether the difference observed between human and fly is dependent on the species studied or the pri-miRNA considered. For this, we investigated whether *Drosophila* pri-miR-14 is also regulated by smORFs. The sequence of this pri-miRNA exhibits six small ORFs (Figure 7a) with, to date, no evidence of translation. To examine a possible role of smORFs on pri-miR-14/miR-14 expression, we constructed vectors expressing pri-miR-14 with the WT or all ATG-mutated smORFs, except for smORF6 that overlaps pre-miR-14 (Figure 7a). Transfection of these pri-miR-14 constructs in *Drosophila* S2 cells revealed that while the expression of both pri-miR constructs was detected (Figure S7) the ATGs-mutated pri-miR-14 construct (MUT) was expressed at higher levels (2.5-fold) than WT (Figure 7b) and yielded higher levels of mature miR-14 (Figure 7c). To characterize the potential translation of pri-miR-14, in vitro translation experiments were performed using constructs in which each smORF, except smORF1 that was excluded in the analysis because of its small size (27 nt), was fused to an HA tag. An efficient translation was observed only for smORF4 (225 nt, Figure 7d). ATG/TAA mutation of this smORF (MUT ORF4) behaved similarly to the ATGs-mutated construct (MUT), expressing higher levels of pri-miR-14 (Figure 7e) and miR-14 (Figure 7f) than WT. The RNA fold prediction tool indicated no impact on pre-miR-14 secondary structure for the two pri-miR-14 mutants (Figure S8). Importantly, overexpression of the smORF4-encoded peptide, miPEP14, in S2 cells had no impact on pri-miR-14 and miR-14 levels (Figure S9)**,** suggesting that smORF4 also regulates pri-miR-14/miR-14 expression in *cis*. We then tested whether smORFs impinge on miR14 activity by generating flies overexpressing WT or mutant pri-miR-14. We focused on the *Drosophila* wing, where miR-14 overexpression reduces wing size [58]. Despite being overexpressed approximately 20-fold (Figure S7), WT pri-miR-14 transfection in S2 cells only caused a small increase of miR-14 levels compared to endogenous levels (NT) (Figure 7c). Accordingly, no significant variation of wing size was observed in flies overexpressing WT pri-miR-14 compared to control wings (white, w) (Figure 7g, right panel). However, wing size was significantly reduced in flies overexpressing the mutant pri-miR-14, highlighting a higher activity of this pri-miRNA compared to WT (Figure 7g), which is consistent with the increased levels of miR-14 processed from this mutant pri-miR-14 (Figure 7c,f). Thus, smORFs can impinge differently on pri-miRNA expression, and this is apparently unrelated to the species studied but rather is dependent on the pri-miRNA considered.

**Figure 8:**
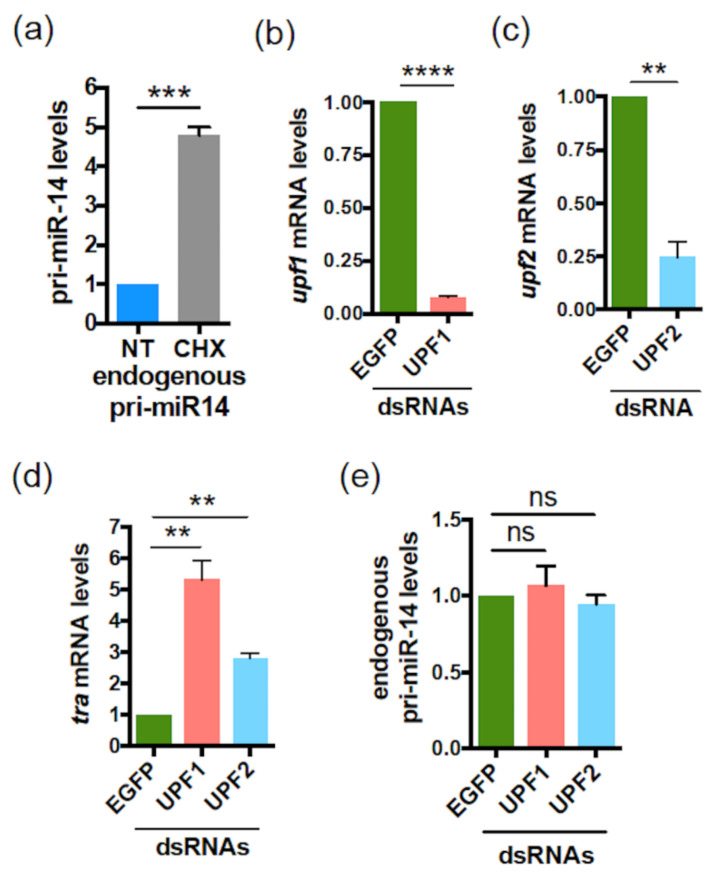
The *Drosophila* pri-miR-14 is regulated by CHX but not by NMD. (**a**) Relative expression levels of pri-miR-14 upon CHX treatment (30 µg/mL for 4 h). Pri-miR-14 levels, determined by qRT-PCR, were normalized to *RP49* and set to 1 for the untreated (NT) cells. Data are means ± S.E.M. *n* = 4. (**b**–**e**) Relative expression levels of *Upf1* (**b**) or *Upf2* (**c**) or *tra* mRNAs (used as positive control) (d) or pri-miR-14 (**e**) in S2 cells transfected with dsRNA directed against EGFP (used as control) of against *Upf1* or *Upf2*. The *Upf1*, *Upf2*, *tra* and pri-miR-14 levels, determined by qRT-PCR, were normalized to *tubulin* and set to 1 for EGFP dsRNA transfected cells. Data are means ± S.E.M. *n* = 4. ** *p* < 0.01, *** *p* < 0.001, **** *p* < 0.0001, ns: not significant.

To test whether translational events are also involved in the regulation of pri-miR-14, as shown for *Human* pri-miR-497 and *Drosophila* pri-miR-8 (Figure 3a,b and Figure 6a), S2 cells were treated or not with CHX. CHX treatment caused an accumulation of endogenous pri-miR-14 (Figure 8a), suggesting again a smORF-dependent regulation mechanism requiring translation. Similar to pri-miR-497, NMD did not appear involved in this mechanism. Indeed, experiments performed using dsRNAs directed against *upf1* (Figure 8b) or *upf2* (Figure 8c), two NMD core components [59], showed no significant variation in endogenous pri-miR-14 levels when these NMD factors were depleted, compared to EGFP dsRNA used as control (Figure 8e). In contrast, *transformer* (*tra*) mRNA, a known NDM substrate [59] accumulated upon *upf1* or *upf2* depletion (Figure 8d), as expected. These results suggest that, similar to pri-miR-497, smORF4 regulates pri-miR-14 expression by an NMD-independent mechanism involving translation.

Altogether these results strongly suggest that smORFs can act in *cis* to fine-tune steady state level expression of pri-miRNAs/miRNAs, either positively or negatively, in *Humans* and *flies*.

## 3. Discussion

In this study, we have identified smORFs as new *cis*-acting elements involved in the regulation of pri-miRNAs and miRNAs expression both in *Humans* and *Drosophila melanogaster* and, to our knowledge, this is the first report of such a function for smORFs. Our results indicate that this function of smORFs is independent of their amino acid sequence. Previously, and in this study, we provided evidence that overexpression of smORF-encoded peptides has no impact on the expression and activity of their pri-miRNAs/miRNAs [36,37], excluding a *trans* effect of the smORFs in the regulation of pri-miRNAs/miRNAs. In contrast, depending on the pri-miRNA studied, smORFs differently influence the expression of pri-miRNAs. Indeed, smORFs have a positive effect on *Human* pri-miR-155 expression, whereas they negatively regulate *Human* pri-miR-497 and *Drosophila* pri-miR-14 expression. The fact that mutation of smORFs affects both the pri-miRNA and miRNA levels, and given that pri-miRNA processing, the first step of miRNA biogenesis, takes place in the nucleus, the suggestion is that smORFs regulate pri-miRNA levels in the nucleus. The mechanism by which smORF51 regulates the expression levels of pri-miR-155 is still unclear. However, for *Human* pri-miR-497 and *Drosophila* pri-miR-8, the substitution of the smORF by another one does not affect pri-miRNA expression levels, indicating that this regulation does not depend on the nucleotide sequence of the smORF, but rather relies upon the presence or absence of a smORF. For *Human* pri-miR-497 and *Drosophila* pri-miR-8 and pri-miR-14, their levels are sensitive to the ribosome-stalling drug cycloheximide, suggesting the involvement of translational events in this smORF-dependent regulation mechanism. This regulation likely occurs with other *Drosophila* pri-miRNAs, because among five further pri-miRNAs analyzed, the levels of four of them were also sensitive to CHX (Figure S10). With the idea that smORFs, present within pri-miRNAs, could be considered by the RNA quality surveillance pathway as premature stop codons (PTCs) in the nucleus, we tested the involvement of NMD, an evolutionarily conserved translation-dependent mechanism that scans mRNAs for PTCs and destroys faulty transcripts. This hypothesis is consistent with several studies providing strong evidence for intranuclear NMD [50,51,52]. Moreover, although it is widely believed that translation occurs only in the cytoplasm, studies have shown that some translation occurs in the nucleus of mammalian cells [60,61,62]. In addition, nascent RNAs bearing premature stop codons (PTCs) are eliminated by a mechanism sensitive to a translation inhibitor, reinforcing the idea that nuclear ribosomes exert RNA surveillance by scanning newly made transcripts, with faulty transcripts being degraded by NMD [60]. In agreement with this idea, other studies have shown that eukaryotic cells can detect ORFs within the nucleus [63]. However, NMD inhibition (*Human* pri-miR-497) or dsRNA against key NMD components (*Drosophila* pri-miR-14) do not indicate an involvement of NMD in the smORF-dependent regulation of these pri-miRNAs. These results contrast with a study identifying Human miRNA host genes (MIRHGs) as NMD targets [26]. However, since these pri-miRNAs encode intronic miRNAs, which are processed from the excised introns, these results were interpreted as translation events of the smORFs present in the cytoplasmic spliced RNAs triggering NMD similarly to smORFs-bearing cytoplasmic lncRNAs [26].

Concerning the *Drosophila* pri-miR-8, ATG-mutation of the smORF213 increases pri-miR-8 levels but decreases miR-8 levels, suggesting that pri-miRNA processing is affected. In agreement with this, mutant pri-miR8 appeared more stable than its WT counterpart and *Drosha* depletion did not result in significant changes in mutant pri-miR-8 levels compared to WT. Since WT pri-miR-8 is sensitive to cycloheximide, this also suggests a link between ribosome and pri-miRNA processing. To date, such a link has never been reported in animals. Interestingly, STV1, a conserved ribosomal protein, binds pri-miRNAs to promote their interaction with the processing complex in *Arabidopsis* [64]. Additionally, a functional interaction has been identified between RNase III and the *E. Coli* ribosome [65], suggesting that a link between ribosomes and RNAse III enzymes is evolutionarily conserved. These results lead us to propose that ribosome interaction with the pri-miRNAs and DROSHA (a RNAse III enzyme) might facilitate pri-miRNA processing. Interestingly, a recent study reported an unconventional role of ribosomes in small-non coding RNA formation [66]. Indeed, ribosomes also mediate PIWI-interacting RNA (piRNA, a class of small non-coding RNA) biogenesis from long intergenic piRNA precursors in mice by first translating the uORFs of piRNA precursors, then translocating them into the uORF downstream regions where the ribosome-protected regions will become piRNAs after endonucleotic cleavage [66].

Montigny et al. 2021 [36] generated flies by knock-in strategy using a specific P landing platform to replace the entire miR-8 locus by the WT or the ATG-mutated smORF213 pri-miR-8 in *Drosophila*. In these transgenic flies, miR-8 was expressed at similar levels to the endogenous miR-8 [36]. Unfortunately, no difference in pri-miR-8 accumulation between the WT and mutant pri-miR-8 was detected in these flies (Figure S11), in contrast to results obtained by overexpression of these two pri-miR-8 in *Drosophila*, revealing that mutant pri-miR-8 accumulates at higher levels than its WT counterpart (Figure S5). This discrepancy can be explained by the fact that this regulation either occurs transiently or takes place only in a few miR-8 expressing cells or is too weak to be significantly detected on endogenous transcripts.

Importantly, this study indicates that smORFs embedded in pri-miRNAs may exhibit dual functions: first they encode functional peptides (called miPEPs for miRNA-encoded peptides) and second, they may act as *cis*-regulatory elements involved in the regulation of pri-miRNA/miRNA expression. Whereas in plants, all miPEPs discovered so far have been found to upregulate their miRNA directly by enhancing the transcription of their associated pri-miRNA [67], this function is apparently not conserved for human and fly miPEPs [36,37]. Instead, in animals the regulation of pri-miRNA/miRNA expression may be carried out by the smORFs themselves, although the precise mechanism still requires further investigation.

Altogether, this study adds a new layer of regulation of miRNA biogenesis. Given the prevalence of smORFs in *Human* and *Drosophila* pri-miRNAs [36,37], it will be very interesting to see how different smORFs influence the levels of different pri-miRNAs, and the proteins involved in this regulation.

## 4. Materials and Methods

### 4.1. Cell Culture, Treatment and Transfections

Human HeLa cells from ATCC were grown at 37 °C with 5% CO2 in DMEM (Gibco, LifeTechnologies, Carlsbad, CA, USA) supplemented with 10% fetal bovine serum and 2 µg/mL penicillin/streptomycin (Sigma-Aldrich, St Louis, MO, USA). *Drosophila* S2 cells were grown in Schneider’s medium (Invitrogen, Waltham, MA, USA) supplemented with 10% fetal bovine serum (Sigma-Aldrich) and 50U/mL penicillin and 50 µg/mL streptomycin (Invitrogen) at 25 °C. Transfection of HeLa or S2 cells with plasmid vectors was performed respectively with JetPrime reagent (Polyplus transfection, Illkirch, France) and FuGene HD transfection Reagent (Promega, Madison, WI, USA) according to the manufacturer specifications. Cycloheximide, Actinomycin D and NMDI14 were purchased from Sigma.

### 4.2. Plasmids and dsRNAs

The pri-miR-155 and pri-miR-497 cloned in the pcDNA3 EGFP, and the luciferase sensors of miR-155 and miR-497 activity cloned in psiCHECK2 dual luciferase reporter vector (Promega) were described previously [37]. The pri-miR-8 cloned into the pUAS-Attb vector (Drosophila Genomics Resource Center, Indiana University, Bloomington, Indiana, USA) and the luciferase sensor of miR-8 activity, pAcLucR3’esg, were described previously [36]. Pri-miR-14 was obtained from PCR amplification of adult fly DNA with primers primiR14sensBglII (5’-tgagcaagatc tcgtttcattcgtcgtcgaacg-3’) and primiR14revXbaI (5’-gatcgactctagactgctgctggaaatatagaggg-3’) and cloned into the pUAS-Attb vector at Bgl2-XbaI restriction sites. PActin-GAL4 vector (pAcGal4, Addgene #24344) was used in S2 cells co-transfection experiments to drive transgene expression from pUAS-attb and to monitor transfection efficiency. The dsRNAs were generated according to [68] using the following primers:

dsRNA-EGFP Forward 5’-TAATACGACTCACTATAGGACGTAAACGGCCACAAGTTC-3’, Reverse 5’-TAATACGACTCACTATAGGTGTTCTGCTGGTAGTGGTCG-3’

dsRNA-upf1 Forward 5’-TAATACGACTCACTATAGGGGATACTCAACCCACGCAGT-3’, Reverse 5’- TAATACGACTCACTATAGGCACTGTTCCTGGTCCCAGTT-3’

ds-RNA-upf2 Forward 5’-TAATACGACTCACTATAGGACCCTTTGTGCTAATGGTGC-3’, Reverse 5’-TAATACGACTCACTATAGGTTAATACGACGGCCAGAAGG-3’

dsRNA-drosha Forward 5’-TAATACGACTCACTATAGGTGCCCAGCTTTTCACTTCTT-3’, Reverse 5’-TAATACGACTCACTATAGGTCCTGAAGACGTTGCTCCTT-3’

### 4.3. Reverse Transcription (RT) and Quantitative Polymerase Chain Reaction (qPCR)

Human cells were harvested 48 h post-transfection and total RNA extractions and RT for quantifications of mRNAs and pri-miRNAs were performed as previously described [37]. Quantifications of mature miRNAs were performed using a stem-loop RT-qPCR procedure according to Varkonyi-Gasic et al. 2007 [69]. The RT stem loop primers were designed according to Tong et al. 2015 [70]. QPCR was performed using the 2X ONEGreen FAST qPCR premix (Ozyme) on a CF × 96 real time system device (BioRad, Hercules, CA, USA) and analyzed with the CFX Manager Software (BioRad), using the 2-∆∆Ct method. Exogenous pri-miRNA levels were normalized to EGFP, and endogenous pri-miRNAs or mRNAs levels were normalized to GAPDH or actin. Mature miRNA levels were normalized to Snord47 or EGFP. Primers for stem loop RT and qPCR were synthesized by Eurofins Genomics (Ebersberg, Germany) and are listed in Table S1.

For fly experiments, total RNA extractions from young adult fruit flies (2–5 days) or S2 cells harvested 48 h post-transfection, RT and qPCR for quantifications of pri-miRNAs and coding genes were performed as previously described [36]. Exogenous pri-miRNA levels were normalized to GAL4 or GAPDH, endogenous pri-miRNAs or coding genes levels were normalized to tubulin or RP49. For quantification of mature miRNAs, a stem loop RT-qPCR procedure [69] was used using RT stem loop primers designed according to Kramer et al. 2011 [71], and the small RNA U14 was used as a reference gene for normalization. Primers for stem loop RT and qPCR were synthesized by Sigma and are listed in Table S2. QPCRs were performed on the LightCycler 480 Instument II (Roche Life Science, Penzberg, Germany) and the RNA abundance of the examined genes was calculated.

### 4.4. Dual Luciferase Reporter Assays

Hela cells were co-transfected with the luciferase sensors of miRNA activity (psiCHECK2) and either the empty vector or pri-miRNAs expressing vectors (pcDNA3 EGFP) as described previously [37]. S2 cells were transfected with the luciferase sensor of miR-8 activity pAcLucR3’esg, the pAcLuc2p vector (constructed by inserting the Luc2P of pGL4.11 vector from Promega into the pAcGAL4 vector), the pAcGAL4 vector and either the empty vector or the pri-miRNAs expressing vectors (pUAS-Attb) as previously described [36]. Transfected cells were lysed 48h after transfection and luciferase activities were measured using the Dual-Luciferase Reporter Assay System (Promega) according to the manufacturer’s instructions, with a luminoskan and the Skanlt^Tm^ software for microplate readers (Thermo Fisher Scientific, Waltham, MA, USA) for the human experiments and a Victor Nivo luminometer instrument (Perkin Elmer, Waltham MA, USA) for the fly experiments. The ratio Renilla luciferase activity (which quantifies the miRNA activity)/Firefly luciferase activity (which monitor the transfection efficiency) was calculated to indicate the activity of the reporter.

### 4.5. Immunoblotting

The smORF sequences cloned into the pF25A ICE T7 Flexi vector (Promega) were translated in vitro using TnT^®^ T7 Insect Cell Extract Protein Expression System (Promega). After addition of Laemmli buffer, translation products were separated on 4–20% SDS-PAGE gels (BioRad) and transferred to 0.1 µm nitrocellulose membrane (Amersham GE Healthcare, UK) according to Lauressergues et al. 2015 [72]. The membrane was blotted overnight at 4 °C with rabbit anti-HA (C29F4) from Cell Signalling Technology (Beverly, MA, USA), washed then incubated at room temperature with HRP-conjugated secondary antibodies (sc-516102, Santa Cruz Biotechnology, CA, USA). After washes, detection was achieved with chemiluminescence detection reagent (Clarity Western ECL substrate, BioRad). Image acquisitions of immunoblots were performed with a ChemiDoc Touch imaging system (BioRad).

### 4.6. Fly Strains and Genetics

*Drosophila stocks* were maintained on standard cornmeal-yeast medium (Dutscher, Bernolsheim, France) and experiments were performed at 25 °C. UAS-pri-miR-8 and UAS-pri-miR-14 transgenic lines were inserted in attP86F site through PhiC31-mediated integration with injections performed by Bestgene Inc (Chino Hills, CA, USA). For analysis of miR-8 activity in imaginal wing discs, the *ptc* GAL4; *tubulin* GFP miR-8 sensor line was crossed either with the UAS mCherry or WT or ATG-mutated UAS pri-miR-8. For the experiments of wing phenotype, UAS-pri-miR-8 and UAS-pri-miR-14 transgenic lines were crossed with MS1096-GAL4 (BDSC:8860). For wing measurements, experiments were performed as previously described [36]. Wings or wing disc images were acquired on a Zeiss Axiozoom stereomicroscope (Zeiss, Germany). Measurements of wing size were performed using IMAGE J software (version 1.49u, Wayne Rasband, Research Services Branch of the NIH’s National Institute of Mental Health, Bethesda, MA, USA).

### 4.7. Statistical Analyses

Statistical analyses were performed using GraphPad Prism software (version 6; GraphPad Software Inc., San Diego, CA, USA). Data are shown as means ± SEM and were considered statistically significant at *p* < 0.05 and *n* represents the number of experiments. For statistical analysis, the Mann-Whitney test or the one sample *t*-test were used. * *p* < 0.05, ** *p* < 0.01, *** *p* < 0.001 and **** *p* < 0.0001, ns: non-significant.

## Data Availability

Not applicable.

## Supplementary Materials

The following supporting information can be downloaded at: https://www.mdpi.com/article/10.3390/ijms23105764/s1.
