# Supplementary material for: Small ORFs as New Regulators of Pri-miRNAs and miRNAs Expression in Human and Drosophila
Source: Int J Mol Sci. 2022 May 20;23(10):5764. doi: 10.3390/ijms23105764 (PMC9144653; doi:10.3390/ijms23105764)
Supplement: Supplementary file 1 [file ijms-23-05764-s001.zip › ijms-1720535 Supplementary.pdf]

Supplementary Figure 1

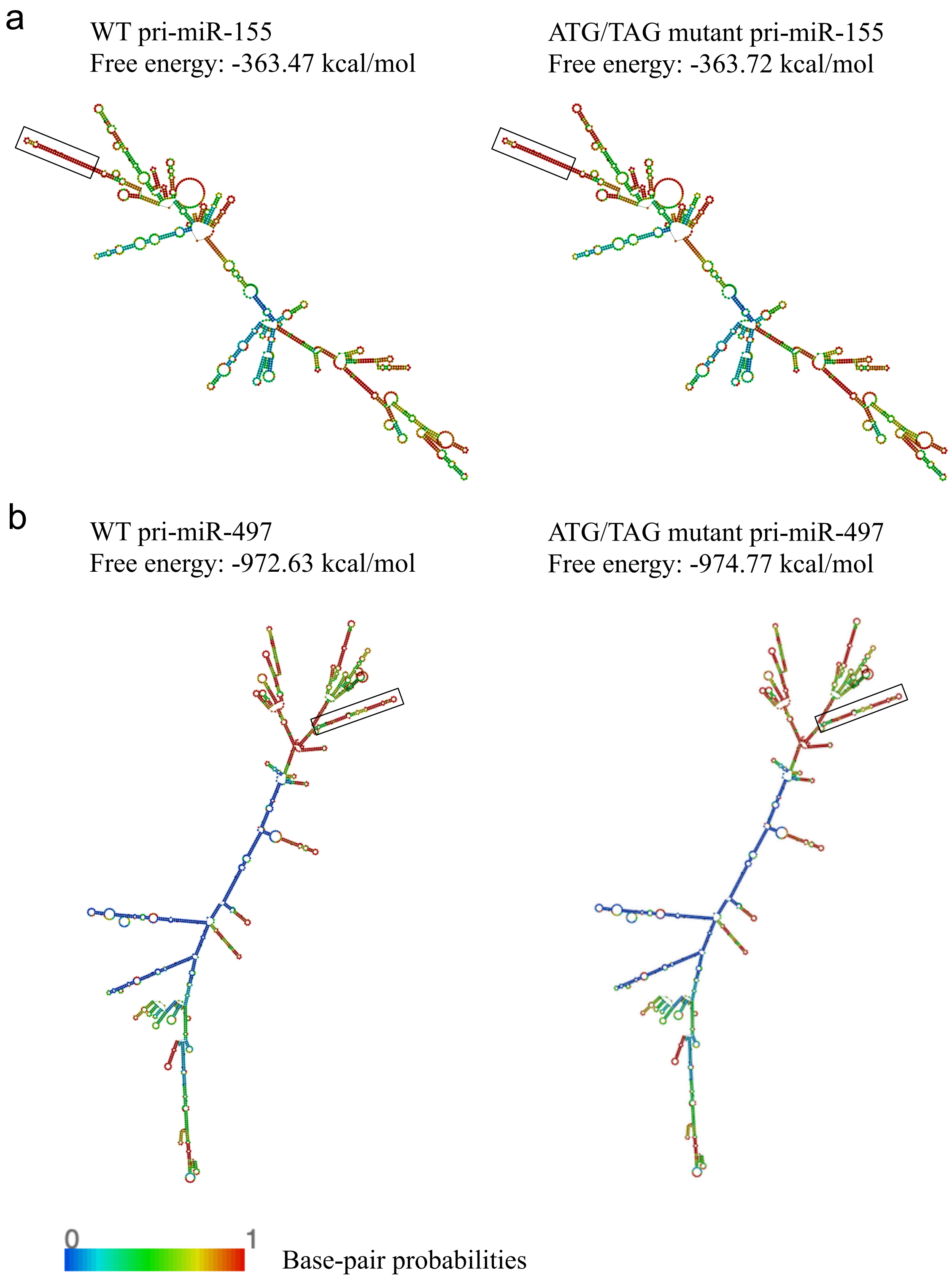

Figure S1: Predicted MFE (minimun free energy) secondary structures for *human* WT and mutants pri-miR-155 (**a**) and pri-miR-497 (**b**), according to RNA Fold software with base-pair probabilities shown, and free energy of the thermodynamic ensemble indicated.The pre-miRNA sequences are boxed.

Supplementary Figure 2

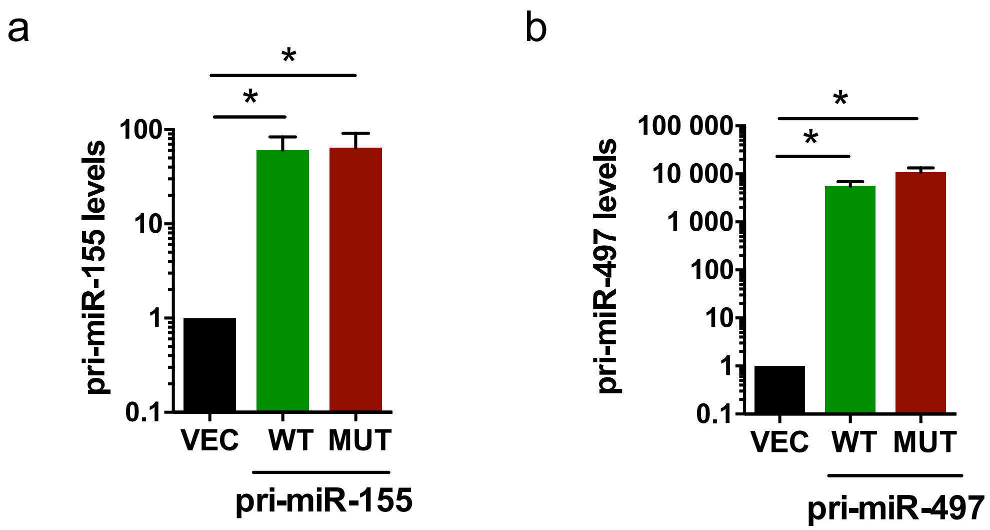

Figure S2: Relative expression levels of WT and mutants pri-miR-155 and pri-miR-497 transfected in Hela cells

Hela cells transfected with either the control vector (VEC) or a vector expressing the WT or ATG-mutated (MUT) pri-miR-155 **(a)** or pri-miR-497 **(b)** were analyzed for pri-miRNA levels by qRT-PCR. Pri-miRNA levels were normalized to GAPDH and set to 1 for the vector transfected cells.
Data are means ± S.E.M. n = 4. * p<0.05

Supplementary Figure 3

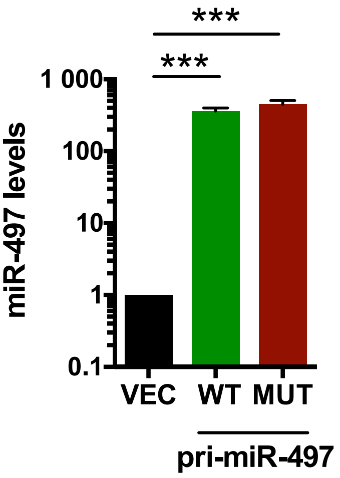

Figure S3: Relative expression levels of miR-497 generated from WT or mutant pri-miR-497 transfected in Hela cells.

Hela cells transfected with either the control vector (VEC) or a vector expressing the WT or mutated (MUT) pri-miR-497 were analyzed for miR-497 levels by qRT-PCR. MiR-497 levels were normalized to Snord47 and set to 1 for the vector transfected cells. Data are means ± S.E.M. *n* = 7.

*** $p<0.0005$

Supplementary Figure 4

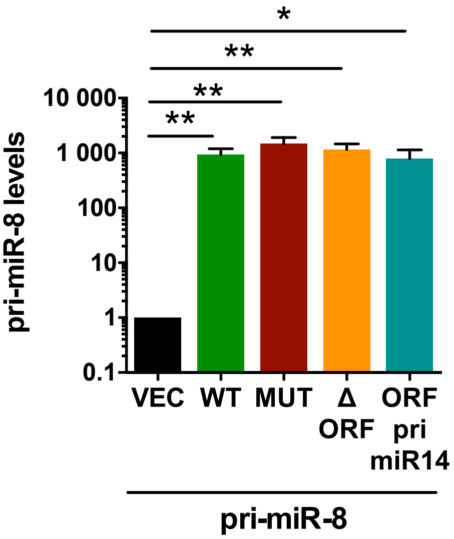

Figure S4: Relative expression levels of WT and mutants pri-miR-8 transfected in S2 cells

S2 cells transfected with either the control vector (VEC) or a vector expressing pri-miR-8 WT or ATG-mutated (MUT), or smORF213 deleted (ΔORF) or pri-miR-8 with smORF213 substituted by smORF225 of pri-miR-14 (ORF pri-miR-14) were analyzed for pri-miR-8 levels by qRT-PCR. Pri-miR-8 levels were normalized to *tubulin* and set to 1 for vector transfected cells. Data are means ± S.E.M. $n = 10$.
* $p<0.05$, ** $p<0.01$, *** $p<0.0005$

Supplementary Figure 5

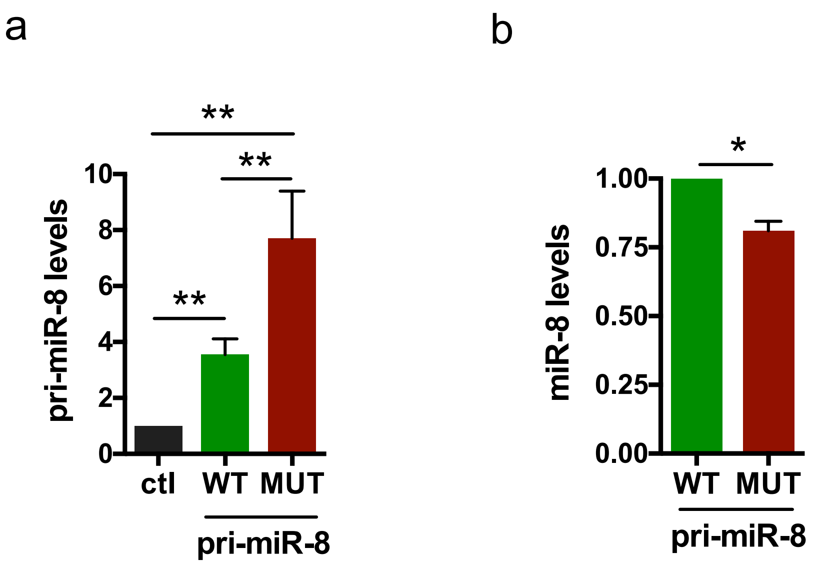

Figure S5: Relative expression levels of WT and mutant pri-miR-8 in fly.
Flies overexpressing the WT or ATG-mutated smORF213 (MUT) pri-miR-8 in miR-8 expression domains, using the miR-8 GAL4 driver (NP5247), were analyzed for pri-miR-8 **(a)** and miR-8 **(b)** levels by qRT-PCR.

(a) Pri-miR-8 levels were normalized to *tubulin* and set to 1 for control flies. Data are means ± S.E.M. n = 6

(b) MiR-8 levels were normalized to *U14* and set to 1 for WT pri-miR-8 flies. Data are means ± S.E.M. n = 3

* p<0.05, **p<0.01

Supplementary Figure 6

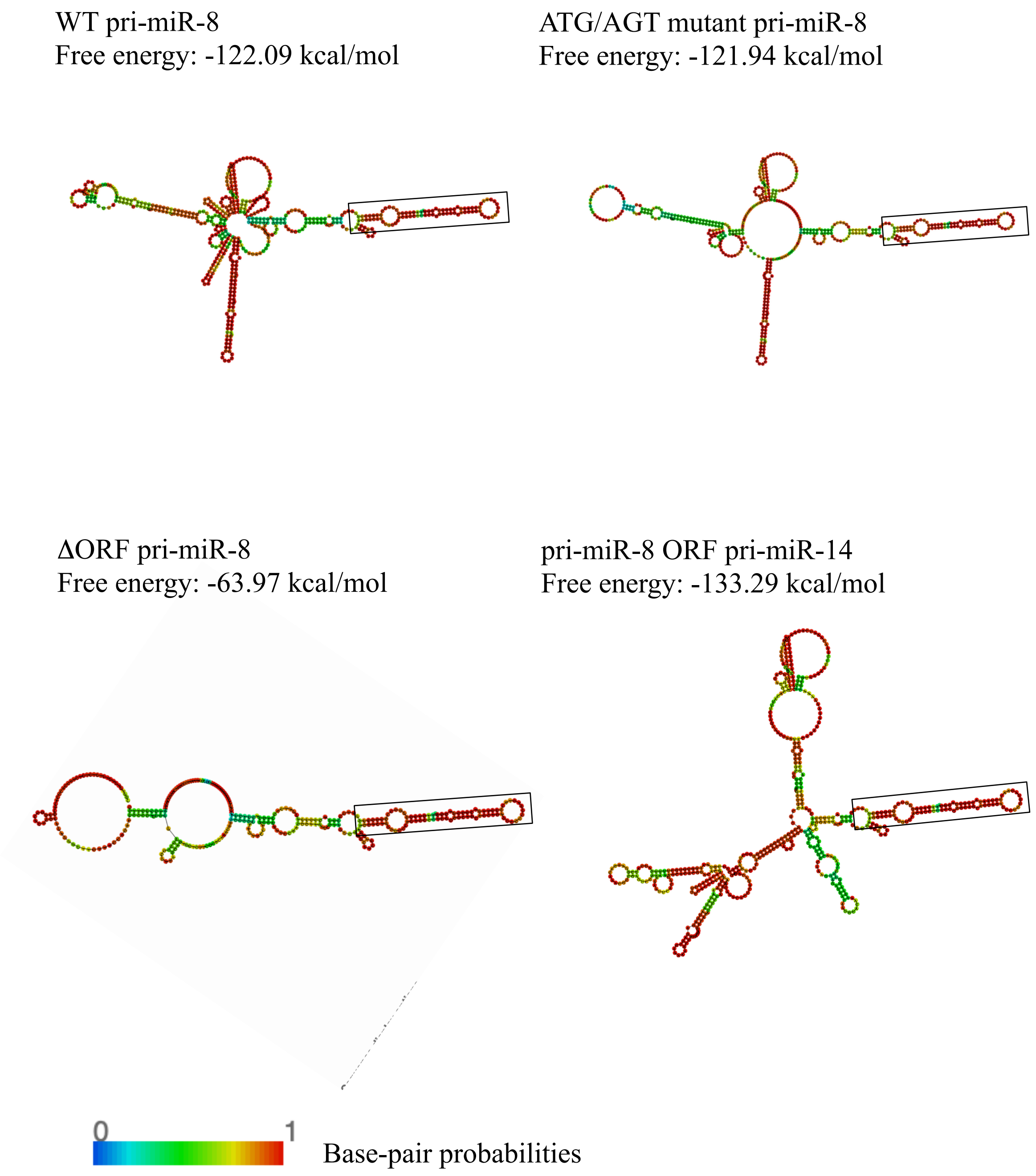

Figure S6: Predicted MFE (minimun free energy) secondary structures for *Drosophila* WT and mutants pri-miR-8 according to RNA Fold software, with base-pair probabilities shown and free energy of the thermodynamic ensemble indicated.
The pre-miRNA sequences are boxed.

Supplementary Figure 7

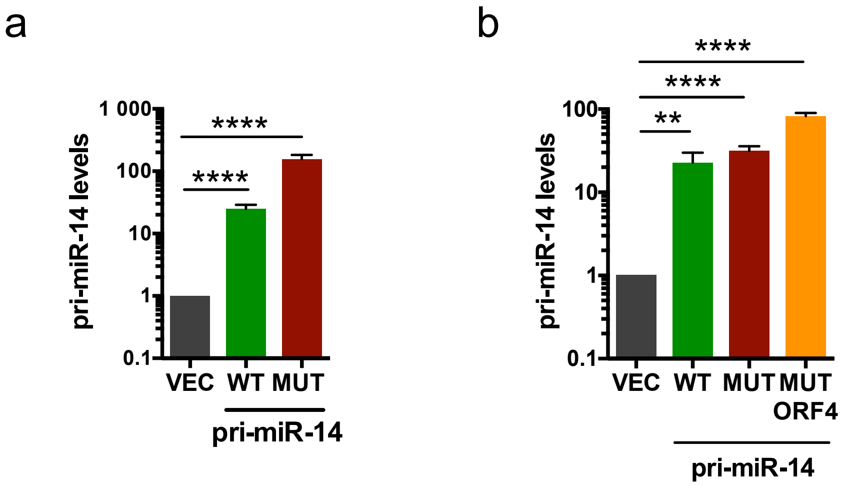

Figure S7: Relative expression levels of WT and mutants pri-miR-14 transfected in S2 cells.

S2 cells transfected with either the control vector (VEC) or a vector expressing pri-miR-14 WT or ATGs-mutated (MUT) **(a, b**) or ORF4 ATG mutated (MUT ORF4) (**b**) were analyzed for pri-miRNA levels by quantitative RT-PCR. Pri-miRNA levels were normalized to *tubulin* and set to 1 for vector transfected cells.

Data are means ± S.E.M. **(a)** $n = 14$, **(b)** $n = 9$.

**p<0.01, **** p<0.0001

Supplementary Figure 8

WT pri-miR-14
Free energy:
-318.82 kcal/mol

MUT ORF4 pri-miR-14
Free energy:
-319.24 kcal/mol

ATGs-mutated pri-miR-14
Free energy:
-296.96 kcal/mol

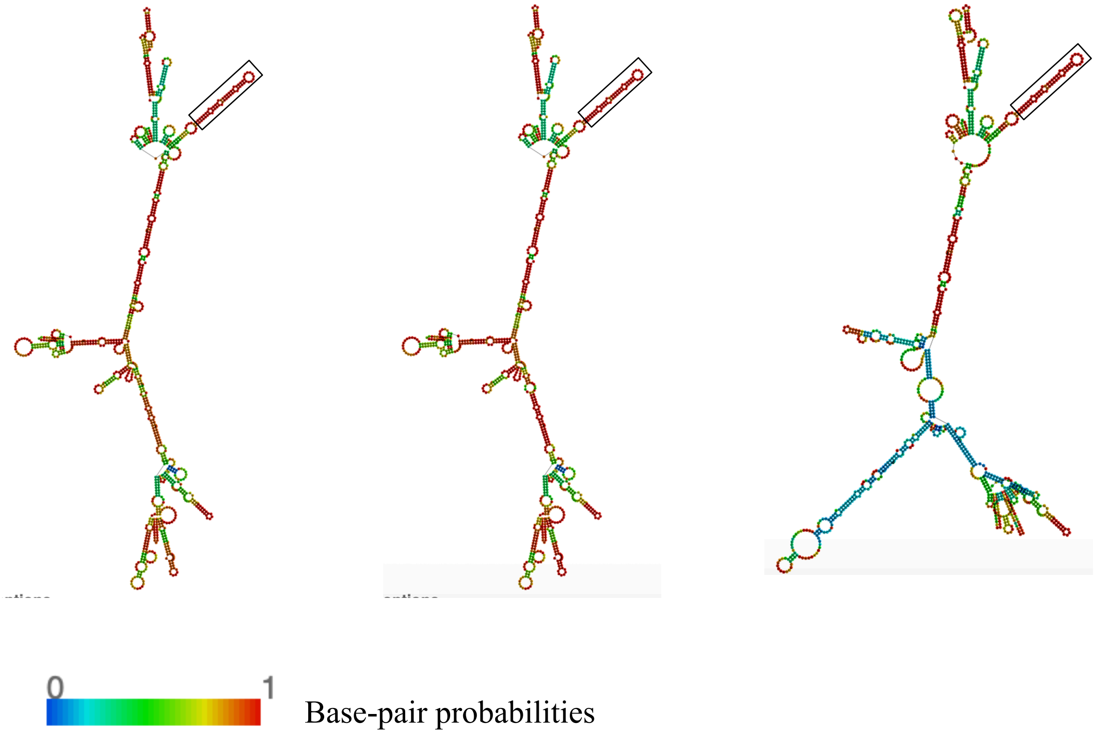

Figure S8: Predicted MFE (minimun free energy) secondary structures for *Drosophila* WT and mutants pri-miR-14 according to RNA Fold software, with base-pair probabilities shown and free energy of the thermodynamic ensemble indicated.

The pre-miRNA sequences are boxed.

Supplementary Figure 9

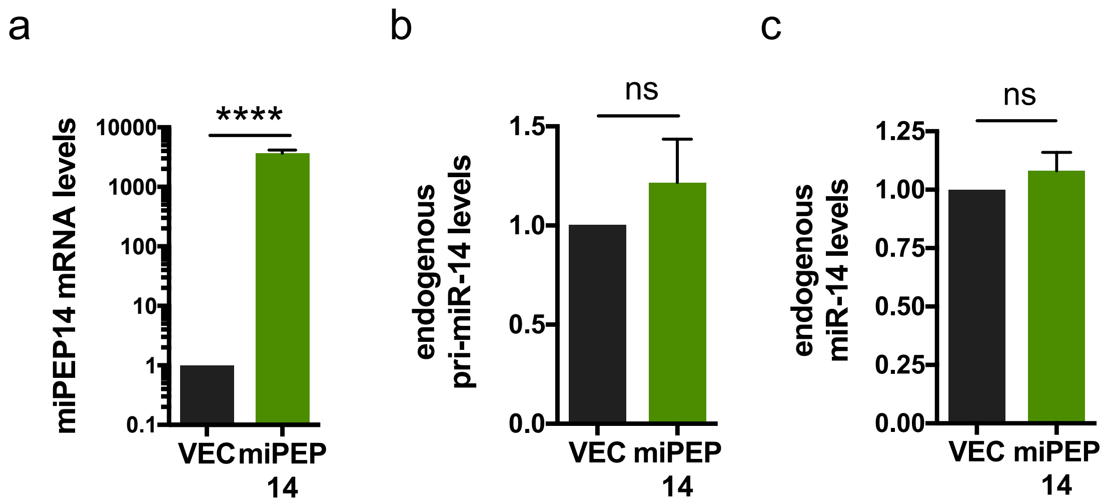

Figure S9: miPEP14 does not regulate pri-miR-14 and miR-14 expression
Relative expression levels of endogenous pri-miR-14 **(b)** or miR-14 **(c)** upon overexpression of miPEP14 in S2 cells. Pri-miRNA and miRNA levels quantified by qRT-PCR were normalized to *tubulin* and *U14*, respectively, and set to 1 for the control vector (VEC) transfected cells. The left panel **(a)** confirms the overexpression of miPEP14 in transfected S2 cells
Data are means ± S.E.M. **(a, b)** n = 8, **(c)** n = 15.
**** $p<0.0001$, ns: not significant

Supplementary Figure 10

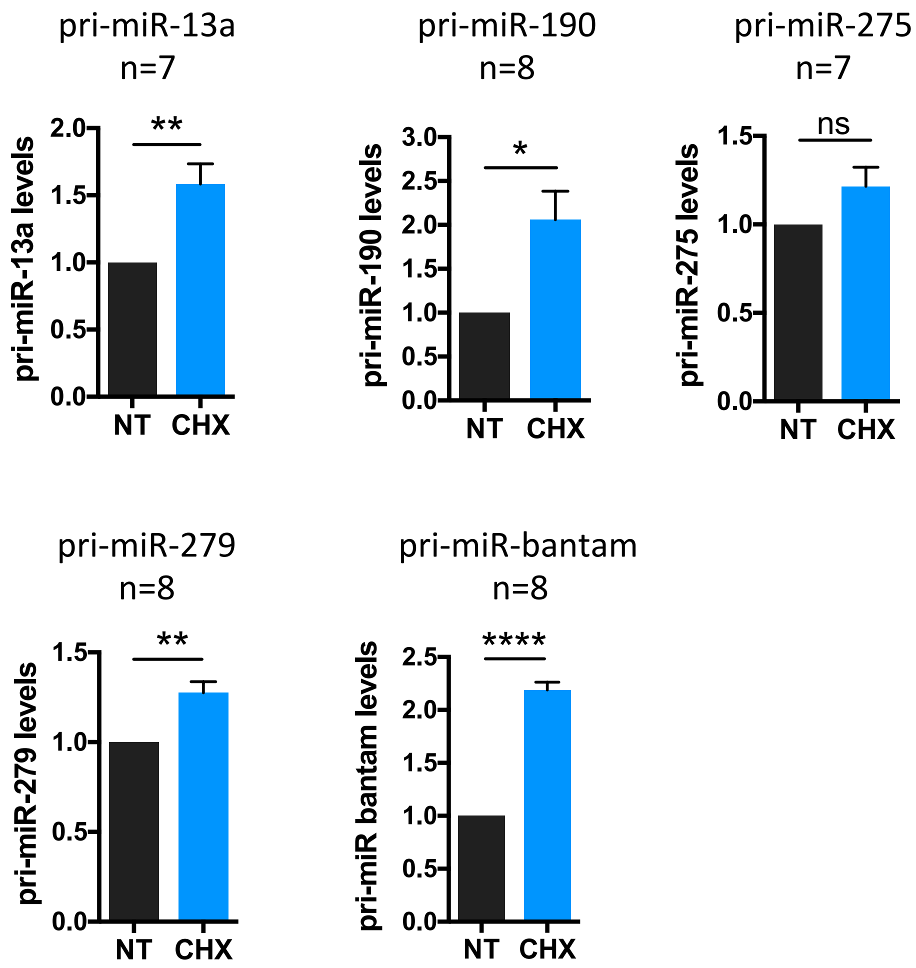

Figure S10: Relative expression levels *of Drosophila* pri-miRNAs in S2 cells upon CHX treatment (30 µg/ml for 4 h). Pri-miRNA levels, determined by q RT-PCR, were normalized to *RP49* and set to 1 for the untreated (NT) cells.
Data are means ± S.E.M.
p<0.05 , **p<0.01, **** p<0.0001, ns: not significant

Supplementary Figure 11

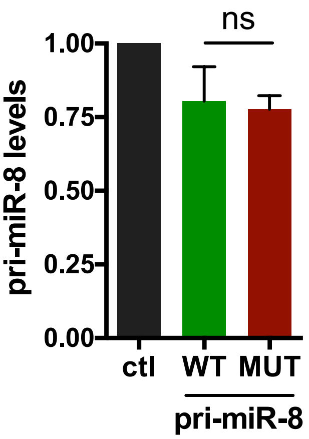

Figure S11: Relative expression levels of WT and mutant pri-miR-8 in Knock-in fly lines.
WT and ATG-mutated pri-miR-8 (MUT) Knock-in fly lines (Montigny et al. 2021) were analyzed for pri-miR-8 levels by qRT-PCR. Pri-miR-8 levels were normalized to *tubulin* and set to 1 for control flies. Data are means ± S.E.M. *n* = 4

Table S1

Sequences of human primers used in stem loop RT and qPCR experiments

FW : Forward, RV : Reverse, SL : stem loop

pri-miR-155	FW<br>RV	5’-GGGAGGATGACAAAGAAGCA-3’<br>5’-TGAACATCCCAGTGACCAGA-3’
pri-miR-497	FW<br>RV	5’-TGGGGTTGGCTTTGTCAGTC-3’<br>5’-CACTATCCTAACGGCTGGGG-3’
EGFP	FW<br>RV	5’-ACCACTACCAGCAGAACACC -3’<br>5’-ATGTGATCGCGCTTCTCGT-3’
GAPDH	FW<br>RV	5’-GAAGGTGAAGGTCGGAGTCA-3’<br>5’-GAAGATGGTGATGGGATTTC-3’
β-actine	FW<br>RV	5’-TGGACTTCGAGCAAGAGATG-3’<br>5’-GAAGGAAGGCTGGAAGAGTG-3’
CDKN1A	FW<br>RV	5’-TCAGAACCCATGCGGCAGCA-3’<br>5’-TGGATGCAGCCCGCCATTAG-3’
miR-497	SL RT<br>FW<br>RV	5’GTCGTATCCAGTGCAGGGTCCGAGGTATTCGCACTGGATACGACACAAACCACAG-3’<br>5’-CGCCAGCAGCACACTGTGG-3’<br>5’-CCAGTGCAGGGTCCGAGGTA-3’
Snord47	SL RT<br>FW<br>RV	5’-GTCGTATGCAGAGCAGGGTCCGAGGTATTCGCACTGCATACGACAACCTC-3’<br>5’-ATCACTGTAAAACCGTTCCA-3’<br>5’-GAGCAGGGTCCGAGGT-3’

Table S2
Sequences of *Drosophila* primers used in stem loop RT and qPCR experiments
FW : Forward, RV : Reverse, SL : stem loop

pri-miR- 8	FW	5'-TCTTACCGGGCAGCATTAGA-3'
	RV	5'-TGATTTGTTTGGATTTTTACGC-3'
pri-miR-14	FW	5'-AACCTATGTGGGAGCGAGAC-3'
	RV	5'-TGTGCCATGATGATACGCCC-3'
pri-miR-13a	FW	5'-CGTAACTCCTCAAAGGGTTGTG-3'
	RV	5'-CGAAACTCATCAAAATGGCTGTG-3'
pri-miR-190	FW	5'-ACTTGCCCTTCAAAACGAACT-3'
	RV	5'-TCAAACATATCTCACTGGAACCATC-3'
pri-miR-275	FW	5'-TCTTCAACTCCAGCCACCAC-3'
	RV	5'-ACTGATTAGCGCGCAAGGTA-3'
pri-miR-279	FW	5'-TGGAATTGGGACTGGAGCTG-3'
	RV	5'-GTGAAACACTGGACCCCCAC-3'
pri-miR-Bantam	FW	5'-AACCGGTTTTCGATTTGGTTTGA-3'
	RV	5'-CATCGGAATGTGGAATGTGGT-3'
tubulin	FW	5'-CGAGACCTACTGCATCGACA-3'
	RV	5'-AGGTCACCGTATGTGGGTGT-3'
GAL4	FW	5'-CCGAATTTGGTGGTCTGTCT-3'
	RV	5'-GCACATCGTCGACAGAAGAA-3'
RP49	FW	5'-AGATCGTGAAGAAGCGCACCAAGC-3'
	RV	5'-GCACCAGGAACTTCTTGAATCCGG-3'
Drosha	FW	5'-ACCATTGAGTACGAAGAGGA-3'
	RV	5'-GACGGTAAGTTGGGCATT-3'
Upf1	FW	5'-TAAAGTGGTGCGTGTTTGCG-3'
	RV	5'-TAGCTCCGAGTTGGTCTCCA-3'
Upf2	FW	5'-TGCCATTAAGTGCCTCAGCA-3'
	RV	5'-CGTGGCTGATAGGAGCTCAG-3'
Tra	FW	5'-GTAGCCAAATCGCGGAACTC-3'
	RV	5'-ATACCAAAGGCTACCACGTCCTC-3'
miPEP14	FW	5'-TATCGGAAGACAAACGGCGA-3'
	RV	5'-TGAGGTCGTCGGAGCTTTTT-3'
miR-8	SL RT	5'-GTCGTATCCAGTGCAGGGTCCGAGGTATTCGCACTGGATACGACGACATC-3'
	FW	5'-GGGGGTAATACTGTCAGGTAAA-3'
	RV	5'-CCAGTGCAGGGTCCGAGGTA-3'
miR-14	SL RT	5'-GTCGTATCCAGTGCAGGGTCCGAGGTATTCGCACTGGATACGACATAGGA-3'
	FW	5'-CGCGC CAGTCTTTTTCT-3'
	RV	5'-CCAGTGCAGGGTCCGAGGTA-3'
U14	SL RT	5'-GTCGTATCCAGTGCAGGGTCCGAGGTATTCGCACTGGATACGACAGTCAG-3'
	FW	5'-GCTTCGGCTTAATGATGGTC-3'
	RV	5'-CCAGTGCAGGGTCCGAGGTA-3'
